# Heat Stress Enhances the Accumulation of Polyadenylated Mitochondrial Transcripts in *Arabidopsis thaliana*

**DOI:** 10.1371/journal.pone.0002889

**Published:** 2008-08-06

**Authors:** Alessio Adamo, John W. Pinney, Andrea Kunova, David R. Westhead, Peter Meyer

**Affiliations:** 1 Center for Plant Sciences, University of Leeds, Leeds, United Kingdom; 2 Institute of Molecular and Cellular Biology, University of Leeds, Leeds, United Kingdom; Purdue University, United States of America

## Abstract

**Background:**

Polyadenylation of RNA has a decisive influence on RNA stability. Depending on the organisms or subcellular compartment, it either enhances transcript stability or targets RNAs for degradation. In plant mitochondria, polyadenylation promotes RNA degradation, and polyadenylated mitochondrial transcripts are therefore widely considered to be rare and unstable. We followed up a surprising observation that a large number of mitochondrial transcripts are detectable in microarray experiments that used poly(A)-specific RNA probes, and that these transcript levels are significantly enhanced after heat treatment.

**Methodology/Principal Findings:**

As the *Columbia* genome contains a complete set of mitochondrial genes, we had to identify polymorphisms to differentiate between nuclear and mitochondrial copies of a mitochondrial transcript. We found that the affected transcripts were uncapped transcripts of mitochondrial origin, which were polyadenylated at multiple sites within their 3′region. Heat-induced enhancement of these transcripts was quickly restored during a short recovery period.

**Conclusions/Significance:**

Our results show that polyadenylated transcripts of mitochondrial origin are more stable than previously suggested, and that their steady-state levels can even be significantly enhanced under certain conditions. As many microarrays contain mitochondrial probes, due to the frequent transfer of mitochondrial genes into the genome, these effects need to be considered when interpreting microarray data.

## Introduction

Polyadenylation of RNAs has a decisive role in the regulation of RNA stability. In eukaryotes, it confers stability for nuclear mRNA, regulates export of processed mRNAs to the cytoplasm and promotes translation initiation [1]; [2]. In bacteria, in contrast, polyadenylation facilitates RNA degradation by attracting the degradosome, a complex containing phosphorylase (PNPase) [3]. As for prokaryotes, polyadenylation of mRNAs in chloroplasts serves as a RNA degradation signal [4] and it promotes mRNA degradation in plant mitochondria [5]; [6]. Plant mitochondrial PNPase degrades rRNA and tRNA maturation by-products, but also removes highly transcribed non-functional RNAs and antisense transcripts, following their polyadenylation. Polyadenylation therefore appears to be part of a RNA turnover system that counterbalances relaxed transcription in plant mitochondria [7].

Polyadenylation has different consequences on the mitochondrial transcripts in different organisms. In human, for example, polyadenylation is required for stabilisation of mitochondrial mRNA [8]. The classical view that polyadenylation of nucleus-encoded RNA is always associated with enhanced RNA stability has also been challenged by the recent discovery of a nuclear exosome system, that includes nuclear polyadenylation and degradation of RNAs transcribed from intergenic regions [9]. It has been suggested that ancient poly(A)-linked degradation functions have been maintained in nuclei, while poly(A) tails acquired a new role in the cytoplasm. It therefore appears that RNA polyadenylation has different signal functions in different RNA control systems, whose effects on the stability of the involved RNAs depend on the origin and structure of the RNA and on the enzyme machinery that is present in subcellular compartments.

The analysis of mitochondrial gene transcripts in plants is complicated by the frequent exchange of DNA between nuclear and mitochondrial genomes. The *Arabidopsis thaliana* ecotype *Columbia-0 (Col)*, for example, contains a 618±42 kb insert of mitochondrial origin (the ‘numt’ insertion) in the pericentric region of chromosome 2 [10]. The insert contains complete copies of the mitochondrial regions A, B, C and D, representing the 367 kb mitochondrial genome, and an additional internal part representing two copies of a complete D region and two copies of a 41 kb section of the A region.

Owing to the presence of the mitochondrial genome in *Col*, microarrays that cover the *Arabidopsis* genome contain probes designed to detect transcripts from the mitochondrial genes. Considering that polyadenylated mitochondrial mRNAs are rare, as they are subject to degradation, one would assume that selective labelling of polyadenylated transcripts would prevent cross-hybridisation of mitochondrial mRNAs in microarray experiments. Much to our surprise, however, when we analysed a group of microarray experiments, we found significant signals for a gene cluster on chromosome 2 that corresponds to the mitochondrial insertion, showing significant correlation with heat shock response genes and up-regulation in response to heat treatment. This observation prompted us to investigate the origin of the corresponding transcripts. We don't find any evidence for expression of the nuclear copies of mitochondrial inserts. Instead, we detect a pool of polyadenylated transcripts of mitochondrial origin that increases after heat treatment.

## Results

### Co-expression of a heat shock transcription factor gene and mitochondrial gene transcripts

**Figure 1:**
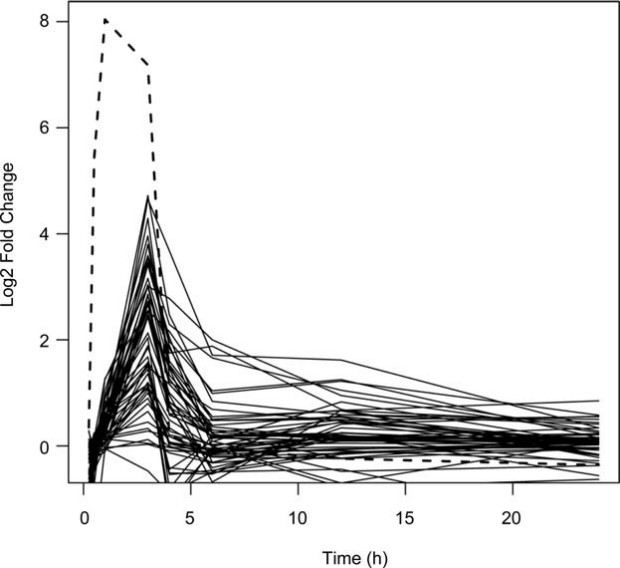
Coordinate increase of numt transcripts after heat treatment. Log_2_ fold change in gene expression between heat treatment and time-matched controls plotted over a 24 hour time course (3h 38°C) heat treatment followed by 21 hours recovery) for the numt insertion genes (black lines) and the heat shock transcription factor (*At2g26150*, dashed line). 0h marks the beginning of the heat treatment.

As part of a general study of gene co-expression and transcriptional regulation, genes showing co-expression with the heat shock transcription factor At2g26150 were investigated in the *Arabidopsis* co-expression tool (ACT). These genes were further clustered with the CliqueFinder tool, and this revealed two main clusters. The first contained a variety of heat and general stress response genes, as would have been expected. Intriguingly, the second comprised 20 genes from the numt insertion. Within this second cluster, transcript levels showed an average pair-wise Pearson correlation of 0.80, and an average correlation of 0.70 with the transcription factor. Full details of the ACT clique clustering analysis are available as supplementary material (Fig. S1). Heat induction of the transcript signals from these genes on the Affymetrix ATH1 microarray was then investigated in a *38°C* heat stress time series experiment. Figure 1 shows the fold change in expression signal in heat stressed plants (shoot tissues) compared to controls over the time course, for the genes of the numt insertion. Many of the genes show a signal that peaks strongly at the 3h time point, subsequently decaying back to normal levels. Of the 53 genes within the insertion for which the ATH1 array has probes, 48 show statistically significant (as judged by a Benjamini-Hochberg corrected p value for differential expression of <0.0001) fold changes at this time point.

### Mitochondrial origin of a heat enhanced polyadenylated transcript

**Figure 2:**
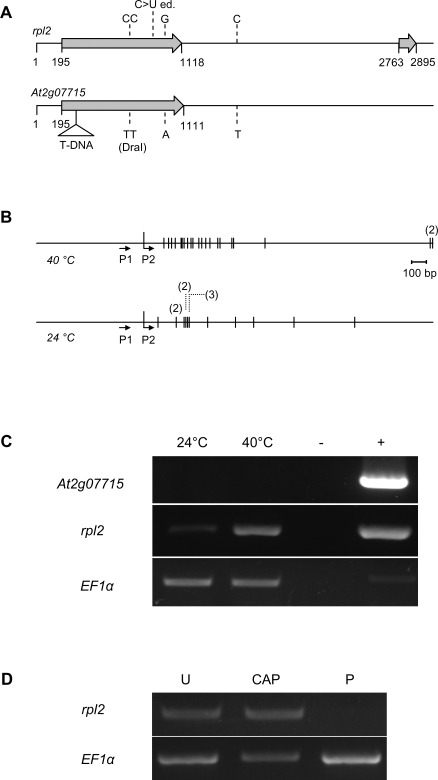
The *Mito1* transcript derives from the mitochondrial copy. (A) Schematic map of the mitochondrial copy of *rpl2*, and its nuclear counterpart integrated into the *Arabidopsis* genome, *At2g07715*. Arrows depict exon ORFs, T-DNA localises the transgene insertion in SALK_143190 Nucleotides mark differences between the two sequences and the C>U editing site of *rpl2*. (B) Vertical bars mark the distribution of polyadenylation sites detected in cDNA copies of *Mito1* transcripts isolated at 40°C or 24°C, respectively. Only four sites were found more than once. P1 and P2 show the localisation of nested primers used for the RT-PCR. Nucleotide positions are labelled with reference to the annotated 5′ end at position 1. The 3′ end of *rpl2* is located at position 3793. [28] (C) RT-PCR primers including the *Dra*I site of the nuclear *Mito1* insertion *At2g07715* (40 cycles) and with primers lacking the *Dra*I site and specific for the mitochondrial *Mito1* gene *rpl2* (32 cycles). Both at 24°C and at 40°C, only *rpl2* specific transcripts could be amplified. EF1α was used to calibrate the amount of cDNA. Genomic wild type DNA was used as positive control (+); (-) indicates control reactions at 40°C without Reverse Transcriptase treatment. (D) RT-PCR analysis of the *Mito1* transcript after sequential treatment with Alkaline Phosphatase (AP), Tobacco Acid Pyrophosphatase (TAP) and Terminator exonuclease (Ter). Lack of signal reduction, compared to the untreated sample (U) after combined treatment with AP, TAP and Ter (CAP) indicates absence of 5′CAP structure. Sensitivity of the transcript to Ter treatment (P) suggests a 5′phosphate structure for the *Mito1* transcript. The Elongation Factor 1 (EF1α) transcript was assayed as a control.

The high similarity between the mitochondrial genome and its genomic insertion makes it difficult to define the origin of these transcripts. We therefore used a T-DNA insertion line (SALK_143190) to tag a mitochondrial model gene in the chromosome 2 cluster, and to sequence the nuclear copy of the gene. We labelled the transcript *Mito1* as it was unclear if it derived from the mitochondrial gene *rpl2* or its nuclear insert *At2g07715*. Sequence comparison of the nuclear insert *At2g07715* with the corresponding mitochondrial gene *rpl2* identified three polymorphisms, one of which created a *Dra*I site in *At2g07715* that was absent in *rpl2* (Fig 2A). An RT-PCR with primers that included the *Dra*I sequence failed to amplify any product, while *rpl2*-specific primers amplified *Mito1* specific transcripts (Fig 2C), suggesting a mitochondrial origin of *Mito1* transcripts. This was confirmed when we cloned cDNAs representing the 3′ part of the *Mito1* transcript, both from material harvested at 24°C and 40°C. All clones matched the mitochondrial *rpl2* transcript, and all except three resembled the unspliced version of the *rpl*2 transcript, with its editing site [11] unchanged. Most cDNAs had different polyadenylation sites, characteristic for the variable polyadenylation of mitochondrial transcripts (Fig. 2B, Fig.S2). Nuclease treatments suggested that the polyadenylated *rpl*2 transcripts did not contain a CAP structure (Fig 2D), as expected for a mitochondrial transcript. All results therefore suggest that the *Mito1* transcripts represent polyadenylated transcripts of the mitochondrial *rpl2* gene.

### The heat-specific increase mainly affects polyadenylated *rpl2* transcripts

**Figure 3:**
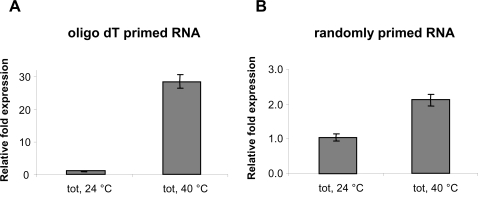
Profile of polyadenylated and total *rpl*2 transcripts levels in total RNA extracts from 10-day-old seedlings. Samples were maintained at 24°C or kept at 40°C for two hours. *Rpl*2 specific values were normalised to *Actin2* transcript levels. Especially in the polyadenylated total RNA fraction, *rpl*2 transcripts are strongly increased after heat treatment.

To differentiate between total and polyadenylated transcripts, we used random primers or oligo dT primers, respectively, to compare *rpl2* transcript levels with and without heat treatment (Fig. 3). In randomly primed RNA pools, *rpl*2 transcript levels increased about 2-fold after heat treatment in total RNA preparations. A similar tendency was observed, although at a more pronounced level, for polyadenylated RNA pools where heat treatment resulted in a 30-fold increase of *rpl*2 transcripts in total RNA preparation. This suggests that the heat-specific enhancement of *rpl*2 transcripts predominantly affects polyadenylated transcripts.

### Recovery from heat-induced transcript enhancement

**Figure 4:**
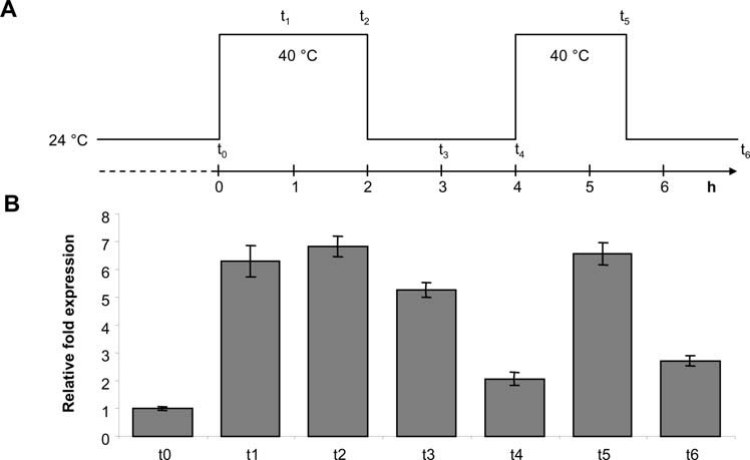
Quantitative kinetics of the accumulation of polyadenylated *rpl*2 transcripts, before and after heat stress in 10-day-old seedlings. (A) Heat application diagram. Samples were taken at time points t0–t6. (B) *Rpl*2 transcript levels at time points t0–t6.

To assess if the accumulation of *rpl*2 transcripts was reversible, we measured transcript levels during and after two heat treatment sessions (Fig. 4). A maximum increase level was reached within one hour of heat treatment. After two hours of a recovery period *rpl*2 transcripts levels had almost reached pre-treatment levels. Very similar increase and recovery effects were detectable during a second heat phase. The data suggest that the temperature effect is reproducible over successive treatment phases, and that transcript levels quickly revert to normal during the recovery phase. The quick reversion of *rpl*2 transcripts levels after the end of the heat treatment implies that polyadenylated *rpl*2 transcripts are quickly degraded once their supply is reduced.

### Turnover rates of *rpl*2 transcripts

**Figure 5:**
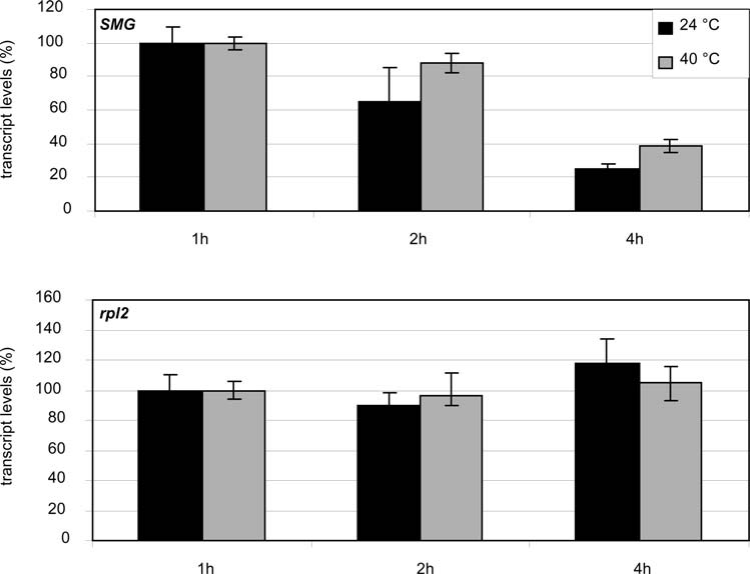
Turnover rates of polyadenylated *rpl*2 transcripts are not significantly affected by heat treatment. When standardized to *EF1α, rpl*2 transcript levels remain stable 1, 2 and 4 hours after Cordycepin treatment of 10-day-old seedlings both at 24°C and 40°C. Transcript levels of the nuclear SMG gene were used as a positive control for the efficiency of Cordycepin treatment.

To assess if the increased temperature affects the stability of polyadenylated *rpl2* transcripts, their level was measured at 24°C and 40°C in the presence of 0.6mM cordycepin [12], which inhibits transcript synthesis. Transcript levels were normalised to *EF1α*. Since *EF1α* is also affected by the treatment, the calculations performed in this way actually show whether the turnover of the tested gene is faster or slower than the turnover of *EF1α*. Over a period of four hours, *rpl*2 transcript turnover rates were comparable to *EF1α* and no changes were detectable for the two temperature treatments with respect to *rpl*2 transcript turnover (Fig. 5), which suggests that heat treatment does not significantly alter the turnover rate of polyadenylated *rpl*2 transcripts.

### Heat-specific increases in other mitochondrial transcripts

**Figure 6:**
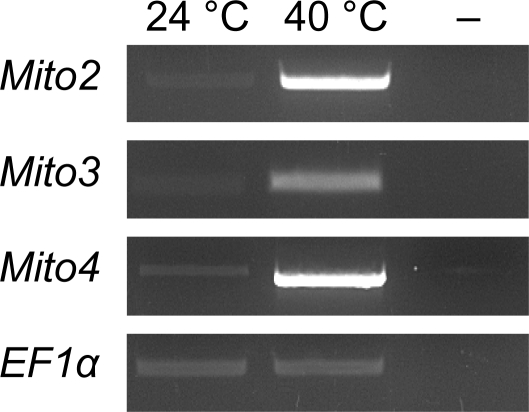
Semi-quantitative RT-PCR of three other polyadenylated mitochondrial transcripts from total RNA preparations reverse transcribed by the oligodT primer at 24°C and 40°C. As for *rpl*2, heat treatment induces an increase in polyA transcript levels. EF1*α* bands were used for sample standardisation. (-) indicates 40°C control reactions without Reverse Transcriptase treatment.

To test if the effects that we observed for the *Mito1* transcript equally apply to other mitochondrial transcripts, we tested the expression of three other genes, *Mito2-4*, in semi-quantitative RT-PCR experiments (Fig 6). For all three genes, we monitored a similar heat response as already observed for *rpl*2 transcripts, which suggests that the effects that we detect for *rpl*2 transcripts equally apply for a number of other mitochondrial transcripts. We also cloned polyadenylation sites of *Mito3* and *Mito4* transcripts (Fig. S3) and observed a very similar variability in polyadenylation as we had previously seen in r*pl2* transcripts

## Discussion

Considering that polyadenylation of mitochondrial transcripts is part of a degradation system, it was surprising to detect positive hybridisation results for so many mitochondrial genes in microarray experiments. It was even more surprising that for a large number of mitochondrial genes, polyadenylated transcripts became more abundant after moderate heat treatment. During evolution, mitochondrial genes have been integrated into nuclear genomes, where they may have developed into functional nuclear copies or from where they may have been lost again [13]. Functional mitochondrial gene transfer into the nucleus is a prominent feature in plants [14] and green algae [15].

Most microarray experiments use the *Columbia-0* ecotype as experimental material, which represents an unusual example for a mitochondrial gene transfer event as it contains a 618±42 kb insert in the pericentric region of chromosome 2 [10]. This insert represents the 367 kb mitochondrial genome with complete copies of the mitochondrial regions A, B, C and D, and an additional internal part that contains two copies of a complete D region and two copies of a 41 kb section of the A region. Comparison of a 262 kb insert of the *Arabidopsis* nuclear mitochondrial DNA (numtDNA) with the mitochondrial DNA (mtDNA) region, revealed a 99.91% identity of the two genomes, and suggested that the mitochondrial DNA transfer into the nucleus occurred about 88,000 (44,000- 176,000) years ago [16]. It is highly unlikely that the genes located on the numtDNA have undergone sufficient adaptation that would allow them to be expressed under the control of the nuclear compartment. Such adaptation would require the development of promoter sequences that allowed transcription by nuclear polymerases, and the establishment of 3′signals that could be recognised by nuclear polyadenylation functions. It was therefore concluded that very little, if any, transcription occurs from numt genes [16].

Our analysis of a model transcript, *Mito1*, which was selected because it allowed us to differentiate between transcripts derived from the mtDNA and the numtDNA, confirms the assumption that the numt gene is not transcribed. The four lines of evidence that point towards a mitochondrial origin of the polyadenylated *Mito1* transcripts are its sequence identity with the mitochondrial gene, the failure to amplify nuclear transcripts, the lack of a CAP structure and the presence of multiple polyadenylation sites, a feature characteristic for mitochondrial transcripts [6]. Enhanced polyA transcript levels quickly revert back to normal levels when the temperature is reduced to 24°C, which suggests that the heat treatment does not cause lasting damage This is also supported by reports about *Arabidopsis* cell cultures being protected from heat-induced cell death at 37°C and only becoming susceptible at 50°C [17]. We therefore have no indications for apoptotic effects from heat stress, which would have been surprising as plants frequently experience periods of high temperatures. A treatment with cordycepin, which inhibits transcription of mitochondrial DNA [18], does not reveal any temperature-specific differences in the turnover rates for the polyadenylated transcripts of the model transcript. This makes it less likely that the heat-specific enhancement in polyadenylated transcripts is the consequence of a reduced efficiency of transcript turnover. We must, however, be careful in our interpretation of cordycepin effects, because cordycepin has been shown to inhibit polyadenylation in some systems [19], and because we could only use a nuclear transcript to confirm the efficiency of the cordycepin treatment. The small but significant increase in randomly primed *Mito1* transcript levels at 40°C suggests that heat stimulates mitochondrial gene transcription. Considering the much larger increase in polyadenylated transcripts, it is tempting to speculate that heat treatment not only increases transcription but also the level of faulty transcript synthesis that leads to enhanced polyadenylation activity.

Three other mitochondrial transcripts show a similar heat-dependent increase of their polyadenylated transcripts, which suggests that the effect is not limited to a single gene but that it affects a number of mitochondrial transcripts. The practical consequence of this effect is that we have to be careful in interpreting the origin of poly(A)-specific transcripts in profiling experiments. In a recent study on chromosome 2 specific transcript variants, in which RNA had been prepared from a variety of tissues subjected to different treatments, including heat, a significant number of transcripts were cloned from mitochondrial insertion genes [20]. Exclusion of 5′phosphorylated transcripts by Terminator nuclease would help to differentiate between nuclear transcripts and polyadenylated transcripts of mitochondrial origin.

## Materials and Methods

### Microarray data analysis

The Arabidopsis co-expression tool (ACT) [21] [22] was used for co-expression analysis of relevant genes over a large database of microarray experiments. The CliqueFinder tool within this software was used to identify groups of genes with mutually high levels of co-expression. The heat stress time series from AtGenExpress (Nover et al. Gene Expression Omnibus (GEO) Accession GSE5628) and the corresponding control time series (Townsend et al. GEO accession GSE5620) were used in detailed investigation of heat induction. These data sets were downloaded from GEO, expression summaries were obtained with the RMA algorithm [23], and differential expression analysis was carried out using linear models in the LIMMA package [24], implemented in the R statistical programming environment.

### Genetic material

A SALK insertion line (ecotype Col-0) with a T-DNA insertion [25] in the coding sequence of *At2g07715* (SALK_143190) was obtained from the Nottingham Arabidopsis Stock Center [26]. Plants carrying the insertion were identified using primers 5′-GGAGAACCTGCGTGCAATCC-3′ and 5′-GCCCTAAAGTATCTTGCCGC-3′. The positive plants were selfed and the homozygosity status of the insertion allele was tested by screening its segregation in the progeny.

Polymorphisms between *At2g07715* and *rpl2* were detected using the primer 5′-TCACACAGTGAATAAGGGCTTAGG-3′ and the primer 5′-GGAGAACCTGCGTGCAATCC-3′ or 5′-ATGAGACCAGGGAGAGCAAGAGC-3′ respectively. The PCR fragments obtained were fully sequenced and compared.

### RT-PCR

RNA was purified with Trizol reagent (Invitrogen) according to manufacturer's instructions. In RT-PCR experiments *Mito1* (*ATMG00560* common mt name *rpl2*; corresponding nu gene *At2g07715*) transcripts were amplified with primers 5′-AGATCCTCGAACCTACCACG-3′ and 5′-GCCCTAAAGTATCTTGCCGC-3′; *Mito2* (*ATMG00513* common mt name *nad5a*; corresponding nu gene *At2g07711*) transcripts were amplified with primers 5′-TGCTCGGTAGTTCCGTAGCAG-3′ and 5′-GGGTGTGCTGTCCCTATTGGGCTG-3′; *Mito3* (*ATMG00370* common mt name *orf199*; corresponding nu gene *At2g07739*) transcripts were amplified with primers 5′-GATAATCAATTCGGTCGTTGTGG-3′ and 5′-ATTGTATGAGGTCTACCCAATGC-3′; *Mito4* (*ATMG01200* common mt name *orf294*; corresponding nu gene *At2g07698*) transcripts were amplified with primers 5′-CAGGAGAATGAGTCCCGCCTCGA-3′ and 5′-GGAAATATTCCCCCATGGCACACC-3′.

In the qRT-PCR experiment *Mito1* transcripts were amplified with primers 5′-CGTCTTCTTCTCTGCCTTCTCCTC-3′ and 5′-TTTTCCTCTCACTTTCTGTCTCATTCG-3′; *Actin2* (*At3g18780*) transcripts were amplified with the primers 5′-CTCAGGTATCGCTGACCGTATGAG-3′ and 5′-CTTGGAGATCCACATCTGCTGGAATG-3′. As a control for the cordycepin treatments, transcripts of the *SMG7a* gene (*At5g19400*) were amplified with primers QSMGF 5′-TTGCTTCTTATTCGAGGGATGAGTTTG-3′ and QSMGR 5′-TCTTGGATGAGTCTTTGGAGGGC-3′.

To initiate reverse transcription, random hexamers were used for random priming and a oligodT primer 5′ GGCCACGCGTCGACTAGTACTTTTTTTTTTTTTTTTT-3′ for polyA-specific transcript priming. The qPCR products were quantified according to the 2(-Delta Delta C(T)) method [27].

### Poly(A) analysis (3′ RACE)

5 µg of total RNA from 10-day-old seedlings was reverse transcribed using Superscript II reverse transcriptase (Invitrogen) according to the manufacturer's instructions using the primer 5′-GGCCACGCGTCGACTAGTACTTTTTTTTTTTTTTTTT-3′. The 3′ end of the *rpl2* transcripts was amplified by semi-nested PCR, using primer 5′-GGCCACGCGTCGACTAGTAC-3′ with primer 5′-GGAGAACCTGCGTGCAATCC-3′ for the primary amplification and with primer 5′-AGCATTCTCTGGGCACATAGG-3′ for the secondary amplification. The PCR product was cloned into the TOPO-TA Vector (Invitrogen) according to the manufacturer's instructions and sequenced. Similarly, the 3′ end of *Mito3* was amplified using primer 5′-GGCCACGCGTCGACTAGTAC-3′ with primer 5′-GATAATCAATTCGGTCGTTGTGG-3′ for the primary amplification and with primer 5′-GGATTTCTGACCACATTCTCC-3′ for the secondary amplification. The 3′ end of *Mito4* was amplified using primer 5′-GGCCACGCGTCGACTAGTAC-3′ with primer 5′-CAGGAGAATGAGTCCCGCCTCGA-3′ for the primary amplification and with primer 5′-AGATCGGTCGAGTGGTCTCAG-3′ for the secondary amplification.

### CAP analysis

15 µg of total RNA obtained from 10-day-old seedlings was split in three aliquots. An aliquot was left untreated (control). An aliquot was treated with Terminator enzyme (Epicentre® Biotechnologies) according to the manufacturer's instructions to selectively digest 5′-monophosphate transcripts. The last aliquot was incubated at 37°C for 30 minutes with Calf Intestinal Alkaline Phosphatase (Promega) in the provided buffer to remove 5′ phosphate groups from transcripts. The RNA was then extracted with phenol:chloroform and precipitated with ethanol. The CAP structure was removed from the RNA with Tobacco Acid Pyrophosphatase (Epicentre® Biotechnologies) according to manufacturer's instructions. After extraction with phenol:chloroform and precipitation the RNA was finally treated with Terminator enzyme (Epicentre® Biotechnologies) according to manufacturer's instructions. The three aliquots were reverse transcribed with Superscript II reverse transcriptase (Invitrogen) using an oligo-dT primer.

## Supporting Information

Figure S1 ACT clique clustering analysis that identifies genes showing co-expression with the heat shock transcription factor At2g26150.
(4.10 MB TIF)

Figure S2 Position and composition of polyA tails detected in rpl2 transcripts isolated at 40°C (underlined) or 24°C, respectively. Nucleotides are numbered according to the transcriptional start site at position 1.
(0.22 MB TIF)

Figure S3 Position and composition of polyA tails detected in orf294 (Mito3) and orf199 (Mito4) transcripts isolated at 40°C (underlined) or 24°C, respectively. Nucleotides are numbered according to the ORF ATG at position 1.
(0.19 MB TIF)
