# Supplementary figures and images for: Heat Stress Enhances the Accumulation of Polyadenylated Mitochondrial Transcripts in Arabidopsis thaliana
Source: PLoS One. 2008 Aug 6;3(8):e2889. doi: 10.1371/journal.pone.0002889 (PMC2483354; doi:10.1371/journal.pone.0002889)

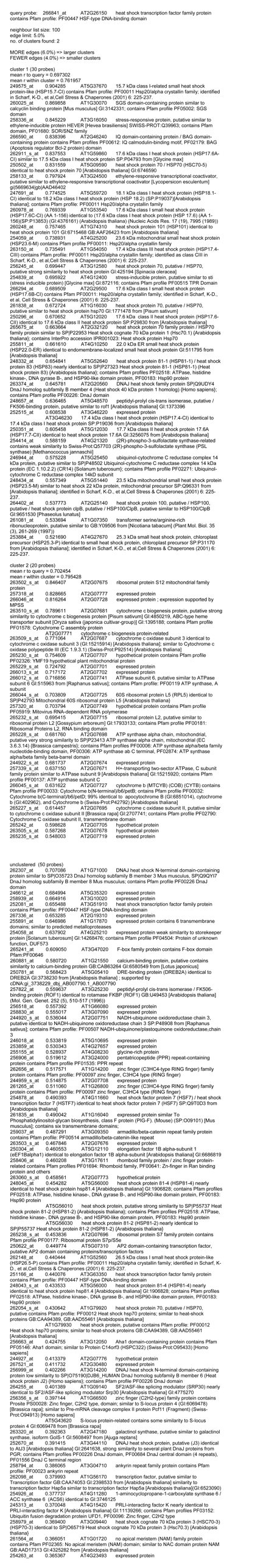

Supplement: Figure S1 — ACT clique clustering analysis that identifies genes showing co-expression with the heat shock transcription factor At2g26150. (4.10 MB TIF) [file pone.0002889.s001.tif]

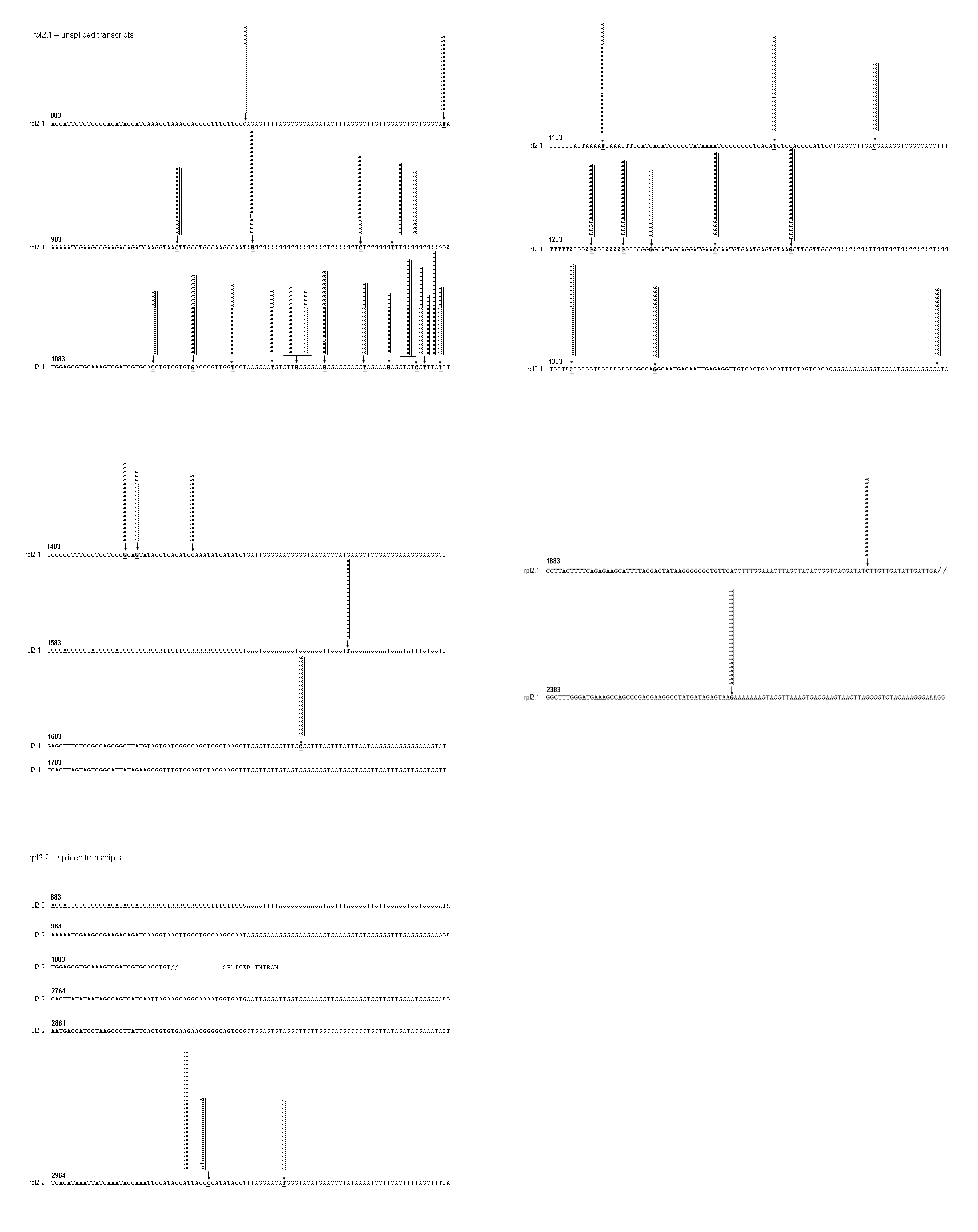

Supplement: Figure S2 — Position and composition of polyA tails detected in rpl2 transcripts isolated at 40°C (underlined) or 24°C, respectively. Nucleotides are numbered according to the transcriptional start site at position 1. (0.22 MB TIF) [file pone.0002889.s002.tif]

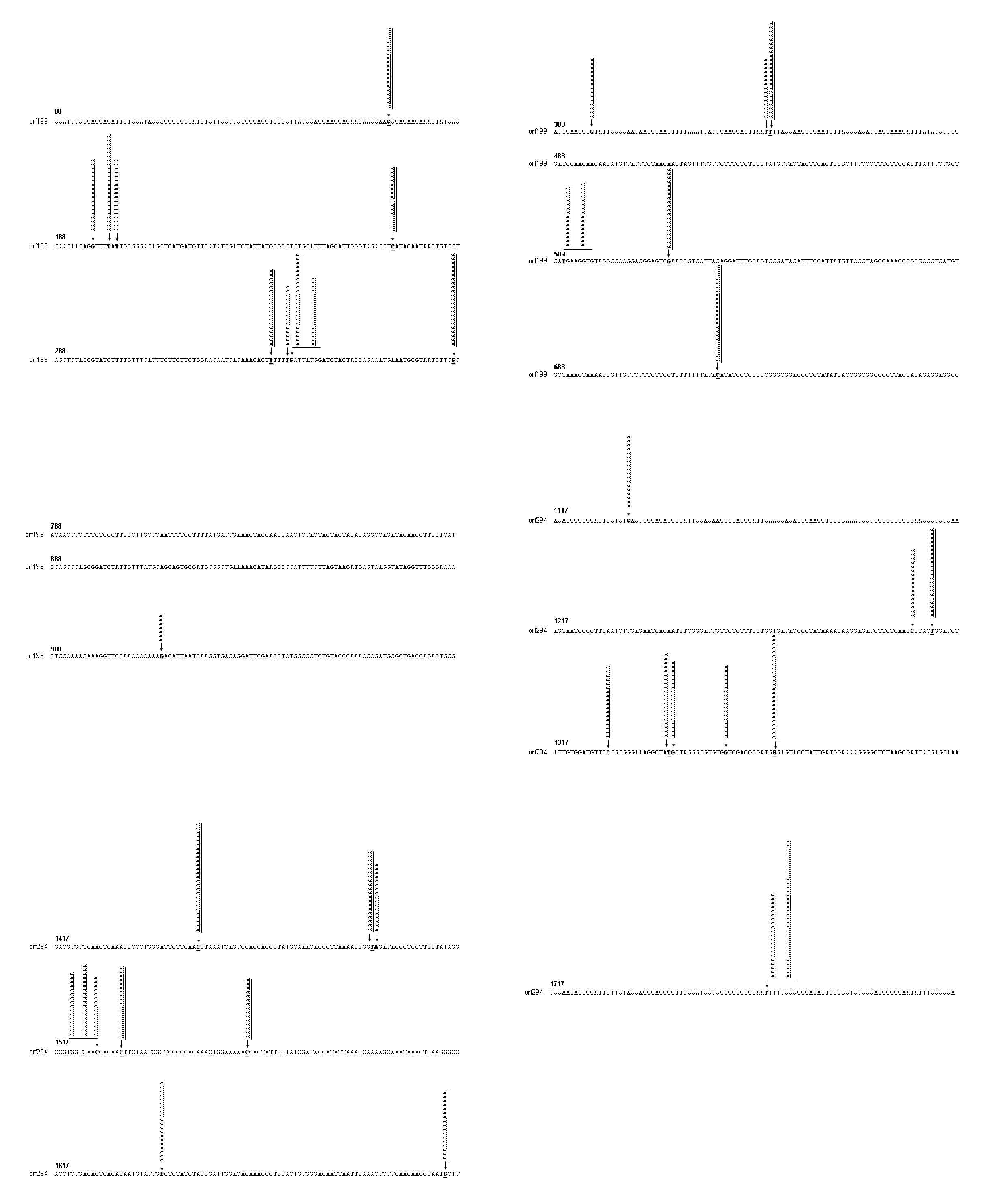

Supplement: Figure S3 — Position and composition of polyA tails detected in orf294 (Mito3) and orf199 (Mito4) transcripts isolated at 40°C (underlined) or 24°C, respectively. Nucleotides are numbered according to the ORF ATG at position 1. (0.19 MB TIF) [file pone.0002889.s003.tif]
